# Transcriptome analysis and immune gene expression of channel catfish (*Ictalurus punctatus*) fed diets with inclusion of frass from black soldier fly larvae

**DOI:** 10.3389/fphys.2023.1330368

**Published:** 2024-01-09

**Authors:** Nithin Muliya Sankappa, Miles D. Lange, Mediha Yildirim-Aksoy, Rashida Eljack, Huseyin Kucuktas, Benjamin H. Beck, Jason W. Abernathy

**Affiliations:** ^1^ Oak Ridge Institute for Science and Education (ORISE), ARS Research Participation Program, Oak Ridge, TN, United States; ^2^ United States Department of Agriculture, Agricultural Research Service, Aquatic Animal Health Research Unit (AAHRU), Auburn, AL, United States; ^3^ U.S. Fish and Wildlife Service, Southwestern Native Aquatic Resources and Recovery Center, Aquatic Animal Health Unit, Dexter, NM, United States

**Keywords:** channel catfish, frass, alternative diets, feed additives, RNA-seq, innate immunity, adaptive immunity, metabolism

## Abstract

The larval waste, exoskeleton shedding, and leftover feed components of the black soldier fly and its larvae make up the by-product known as frass. In this study, we subjected channel catfish (*Ictalurus punctatus*) to a 10-week feeding trial to assess how different dietary amounts of frass inclusion would affect both systemic and mucosal tissue gene expression, especially in regard to growth and immune-related genes. Fish were divided in quadruplicate aquaria, and five experimental diets comprising 0, 50, 100, 200, and 300 g of frass per kilogram of feed were fed twice daily. At the end of the trial, liver, head kidney, gill, and intestine samples were collected for gene expression analyses. First, liver and intestine samples from fish fed with a no frass inclusion diet (control), low-frass (50 g/kg) inclusion diet, or a high-frass (300 g/kg) inclusion diet were subjected to Illumina RNA sequencing to determine global differential gene expression among diet groups. Differentially expressed genes (DEGs) included the upregulation of growth-related genes such as glucose-6-phosphatase and myostatin, as well as innate immune receptors and effector molecules such as toll-like receptor 5, apolipoprotein A1, C-type lectin, and lysozyme. Based on the initial screenings of low/high frass using RNA sequencing, a more thorough evaluation of immune gene expression of all tissues sampled, and all levels of frass inclusion, was further conducted. Using targeted quantitative PCR panels for both innate and adaptive immune genes from channel catfish, differential expression of genes was identified, which included innate receptors (TLR1, TLR5, TLR9, and TLR20A), proinflammatory cytokines (IL-1β type a, IL-1β type b, IL-17, IFN-γ, and TNFα), chemokines (CFC3 and CFD), and hepcidin in both systemic (liver and head kidney) and mucosal (gill and intestine) tissues. Overall, frass from black soldier fly larvae inclusion in formulated diets was found to alter global gene expression and activate innate and adaptive immunity in channel catfish, which has the potential to support disease resistance in this species in addition to demonstrated growth benefits.

## 1 Introduction

The aquaculture sector has to become more sustainable and globally competitive through the advancement of knowledge on fish nutritional requirements and through the continuous search for sustainable raw materials that can solve strategic problems in fish nutrition (Yildirim-Aksoy et al., 2020a; Yildirim-Aksoy et al., 2020b; Mohan et al., 2022). During the last few decades, significant efforts have been made toward the development of less expensive and more sustainable alternative protein and lipid sources with respect to fish meal (FM) and fish oil (FO), without compromising fish welfare and the overall nutritional value of its diet (Belghit et al., 2019; Fawole et al., 2021; Weththasinghe et al., 2021; Zarantoniello et al., 2022). Plant-derived ingredients, microalgae and microbial biomass, terrestrial animal by-products, and insects have been utilized as an alternative for FM and FO (Zarantoniello et al., 2019; Mohan et al., 2022). Insects as alternative feed ingredients for aquafeed formulations have been previously investigated in herbivorous/omnivorous fish and are considered one of the most promising cost-effective, sustainable, and alternative ingredients to replace FM and FO in fish diets. In fact, besides their presence in the natural diet of both freshwater and marine fish species, insects are characterized as having a proper nutritional profile (especially in terms of amino acids, vitamins, and minerals) for fish and being environmentally friendly in aquaculture (low energy and water consumption, and no arable lands are required) (Nyakeri et al., 2017; Swinscoe et al., 2019; Xu et al., 2020; Mohan et al., 2022; Yildirim-Aksoy et al., 2022). Furthermore, the majority of insect larvae can grow on low-quality organic wastes so that the reuse of the remaining organic substrate results in efficient bioconversion and the larvae or prepupae can have enriched proteins, lipids, minerals, and vitamins (Rana et al., 2015; Yildirim-Aksoy et al., 2020a; Yildirim-Aksoy et al., 2020b; Xu et al., 2021; Mohan et al., 2022). Additionally, many insect species have shown an inhibitory effect on fungal and bacterial growth such that their dietary inclusion may increase the shelf life of feed and also provide beneficial effects on the gut of fish (Sheppard et al., 2002; Van Huis et al., 2013; Hounmanou et al., 2018; De Silva et al., 2019; Hoc et al., 2019; Koutsos et al., 2022; Hasan et al., 2023).

Over the last decade, interest in incorporation of insects in fish diet formulations has significantly increased. As a result, several studies have been conducted on various fish species, such as carnivores, herbivores, and omnivores, in utilizing feeds containing insects. Among the insects, the black soldier fly (BSF) (*Hermetia illucens*, Linnaeus 1758, Diptera, Stratiomyidae family) and its larvae, which are native to the tropical, subtropical, and warm temperate areas of Southern United States (three generation cycles per year) have been shown to be a good replacement of FM and FO (Hoc et al., 2019). In this regard, feed incorporated with different combinations of BSF led to the best overall response in terms of growth and health in trout (Huyben et al., 2019; Fawole et al., 2021; Bolton et al., 2021; Caimi et al., 2021; Hoc et al., 2021; Hwang et al., 2021; Melenchón et al., 2021; Randazzo et al., 2021; Cho et al., 2022; Liu et al., 2022; Ratti et al., 2023), salmon (Belghit et al., 2019; Li et al., 2019; Weththasinghe et al., 2021), clownfish (Vargas-Abúndez et al., 2019), zebrafish (Zarantoniello et al., 2018; Zarantoniello et al., 2019), seabass (Wang et al., 2019; Moutinho et al., 2021), tilapia (Yildirim-Aksoy et al., 2020b; Tippayadara et al., 2021; Agbohessou et al., 2022), catfish (Xiao et al., 2018; Adeoye et al., 2020; Yildirim-Aksoy et al., 2020a; Sudha et al., 2022), and shrimp (Cummins Jr et al., 2017; Yildirim-Aksoy et al., 2022).

The largest aquaculture sector in the United States is farm-raised channel catfish, *Ictalurus punctatus*. One problem faced by the channel catfish industry is related to FM and FO in aquafeeds, as they are limiting and increasing less economical and sustainable for the future (Yildirim-Aksoy et al., 2020a). Despite the advancement in using BSF as an alternative to FM and FO, as compared to other species, studies related to the immune response, gut microbiome, and transcriptomes in the channel catfish are scarce (Mohan et al., 2022). Immune-related gene expression studies were conducted in several species where BSF was included in formulated feeds. In rainbow trout, significant differential expression of genes such as IL-1β; IL-10, TGF-β, COX-2, and TCR-β was observed but black soldier fly larvae (BSFL) diets did not induce any inflammation (Gaudioso et al., 2021). In addition, myogenesis-related gene expression experiments conducted in zebrafish show that BSFL meal may increase growth without having any negative consequences. Similar to rainbow trout, no evidence of inflammation in the gut intestinal parameters was found, and there were no appreciable alterations in stress and immunological indicators (Zarantoniello et al., 2019; Mohan et al., 2022). Several other studies have convincingly examined the effects of BSL in addition to the diets of other commercially reared fish species. In an attempt to determine the effects of FM replacement with defatted BSF in Jian carp, significant upregulation in the relative gene expression of peroxisome proliferator-activated receptor (PPARα) and heat shock proteins (HSP70) was observed (Li et al., 2017; Zhang et al., 2017; Mohan et al., 2022). Although BSFL meal supplementation in Nile tilapia did not affect the innate immunity and growth (Agbohessou et al., 2022), BSFL-supplemented meals as an alternative to FM in juvenile seabass substantially increased cytokine expression and mucin production in the gut and skin and exhibited no detrimental effects on development (Hender et al., 2021). Furthermore, according to Chaklader et al. (2019), *H. illucens* larval diet coupled with poultry by-product meal increased the growth of seabass and considerably elevated immune gene expression in the head kidney.

The majority of these studies are designed to determine the effects of dietary supplementation with BSL and report various immune-related gene activities. However, tissue- or organ-specific immune gene expression patterns, the global effects of any changes in expression levels of these genes, and interactions among adaptive and innate immune factors in response to dietary supplementation with BSL are still not clearly understood in channel catfish. The main goal of using BSFL meal is to find a viable replacement for FM that will allow for sustainable development without impeding fish growth. In addition to benefits to growth and development, the use of BSF as a replacement or additive to fish feed is also used to assess the immune-related gene modulation that supports fish systemic and mucosal immunity during pathogen invasion (Mohan et al., 2022). In channel catfish, multi-organ (especially the liver and intestine)–specific transcriptomic and immune-related gene expression studies in response to the addition of frass from BSFL are lacking. The main goal of this study was to assess the effects of dietary inclusion of frass from BSFL, *H. illucens*, at different concentrations to replace FM on both transcriptomics and immune-related gene expression at the systemic (head kidney and liver) and mucosal (gill and intestine) levels in channel catfish.

## 2 Materials and methods

### 2.1 Experimental fish

Fingerlings of the Marion strain channel catfish, *I. punctatus*, were spawned and maintained at the USDA-ARS, Aquatic Animal Health Research Laboratory (Auburn, AL, United States) on commercial fry and fingerling diets and were acclimated to the experimental basal diet for 2 weeks before stocking. At the end of the acclimation period, fish (average weight of 5.24 ± 0.04 g) were randomly stocked into 20, 110 L aquaria at a density of 50 fish per aquarium. The aquaria were supplied with flow-through dechlorinated, heated (28°C) city water with a flow rate of approximately 0.7 L/min. Water was continuously aerated using air stones. Water temperature and dissolved oxygen in three randomly chosen aquaria were measured once every other day in the mornings, using a YSI model Pro DO meter (Yellow Springs Instrument, Yellow Springs, OH, United States). During the trial, the water temperature averaged 26.8°C ± 1.12°C, and the dissolved oxygen averaged 6.35 ± 0.53 mg/L. The photoperiod was maintained at a 12:12 h light/dark schedule.

### 2.2 Experimental diets and feeding trial

A nutritionally complete, practical basal diet was formulated to contain approximately 31.5% crude protein and 6.2% lipid based on feedstuff values reported in NRC (1993) (Table 1). Five diets containing frass (0, 5, 10, 20, and 30%) as partial replacements of a combination of soybean meal, wheat short, and corn meal on an equal protein basis were prepared. Frass from BSF, *H. illucens*, fed on distillers' dried grain with solubles (DDGS), was donated from EnviroFlight LLC, Yellow Springs, OH, United States. Carboxymethyl cellulose (CMC) was added to all diets as a binding agent. Dry ingredients were thoroughly mixed for 10 min in a Hobart mixer before oil was added. After the oil was diffused, approximately 300 mL of deionized water per kilogram of the diet was added. The moist mixture was extruded through a 3-mm diameter die in a Hobart meat grinder. The resulting moist pellets were air-dried at room temperature to a moisture content of approximately 10%. Pellets were ground into small pieces, sieved to obtain approximate sizes, and stored frozen in plastic bags at −20°C until fed. Fish in four randomly assigned aquaria were fed with one of the five experimental diets twice daily (between 0730 and 0830 h and 1500 and 1600 h) to apparent satiation for 10 weeks (Yildirim-Aksoy et al., 2020a).

**TABLE 1:** Percentage composition of experimental diets.

	Experimental diets (%)^a^				
	1	2	3	4	5
Menhaden fish meal	8	8	8	8	8
Soybean meal	45	44	43	41	39
Frass		5	10	20	30
Wheat short	24	20.4	16.9	9.8	2.5
Corn meal	14	13.8	13.5	13	12.75
Corn oil	4	3.8	3.59	3.18	2.75
Dicalcium phosphate	1	1	1	1	1
CMC	3	3	3	3	3
Vitamin premix^b^	0.5	0.5	0.5	0.5	0.5
Mineral premix^c^	0.5	0.5	0.5	0.5	0.5

CMC, carboxymethyl cellulose. Frass is a by-product of the black soldier fly (*Hermetia illucens*) larva meal industry.

^a^ Diets 1, 2, 3, 4, and 5 contained 0, 5, 10, 20, and 30% frass, respectively.

^b^ Vitamin premix, diluted in cellulose, provided by following vitamins (mg/kg diet): vitamin A (520,400 IU/g), 7.7; vitamin D3 (108,300 IU/g), 18.5; vitamin E (250 IU/g), 200; vitamin K, 10; thiamin, 10; riboflavin, 12; pyridoxine, 10; calcium pantothenate, 32; nicotinic acid, 80; folic acid, 2; vitamin B12, 0.01; biotin, 0.2; choline chloride, 400; and L-ascorbyl-2-polyphosphate (35% vitamin C activity), 172.

^c^ Trace mineral premix provided by following minerals (mg/kg diet): zinc (as ZnSO_4__7H_2_O), 150; iron (as FeSO_4__7H_2_O), 40; manganese (as MnSO_4__7H_2_O), 25; copper (as CuCl_2_), 3; iodine (as Kl), 5; cobalt (as CoCl_2__6H_2_O), 0.05; and selenium (as Na_2_SeO_3_), 0.09.

### 2.3 Collection of tissues from channel catfish fed with frass inclusion diets

After the 10-week experiment to evaluate the dietary inclusion of frass in five experimental diets, channel catfish from each treatment (diet) were euthanized by an overdose of buffered MS-222, and the head kidney, liver, intestine, and gills were collected and stored in an RNA stabilization solution (RNA*later*, Thermo Fisher Scientific, Waltham, MA) and stored at −80°C until required. In total, four fish from each aquarium from each of the experimental diets were collected and sampled, and their tissues were pooled together at the end of the trial. Liver and intestine samples were collected for RNA sequencing, while additional samples of the head kidney and gills were collected for quantitative PCR analyses.

### 2.4 RNA sequencing of channel catfish fed with frass inclusion diets

Intestine (n = 16 in pools of 4) and liver (n = 16 in pools of 4) tissues from fish at the end of the 10-week trial from three experimental diets [control (0 g/kg), low frass (50 g/kg), and high frass (300 g/kg)] were used to make libraries for RNA sequencing. Total RNA was extracted from each sample after homogenizing it in a FastPrep-96™ bead beating grinder (MP Biomedicals, Santa Ana, CA) followed by the Qiagen RNeasy^®^ Plus Mini Kit (Germantown, MD) according to the manufacturer’s protocol. Total RNA samples were sent to a service provider (Azenta Life Sciences, South Plainfield, NJ) for library preparation with poly(A) selection and Illumina sequencing. There, RNA samples were quantified using a Qubit 2.0 Fluorometer (Life Technologies, Carlsbad, CA), and RNA integrity was checked using an Agilent TapeStation 4200 (Agilent Technologies, Palo Alto, CA). RNA sequencing libraries were prepared using the NEBNext Ultra II RNA Library Prep Kit for Illumina using the manufacturer’s instructions (New England Biolabs, Ipswich, MA). The sequencing libraries were validated on the Agilent TapeStation (Agilent Technologies, Palo Alto, CA) and quantified by using a Qubit 2.0 Fluorometer (Thermo Fisher Scientific, Waltham, MA) as well as by quantitative PCR (KAPA Biosystems, Wilmington, MA, United States). Libraries were then sequenced on an Illumina HiSeq 4000 according to the manufacturer’s instructions using a 2 × 150 bp paired end (PE) configuration. A total of 24 samples (n = 12 intestine; n = 12 liver) were sequenced to include no frass control diet (n = 4 each tissue), low-frass inclusion of 50 g/kg (n = 4 each tissue), and high-frass inclusion of 300 g/kg (n = 4 each tissue).

### 2.5 Transcriptome analysis for the channel catfish

Raw, demultiplexed reads at a minimum of 25M PE reads/sample were delivered by the service provider. Bioinformatics was performed in-house within the OmicsBox software (OmicsBox, 2019) and R-Bioconductor packages. Raw FASTQ files were initially preprocessed for quality control (QC) using FASTQC (Andrews, 2017) and Trimmomatic (Bolger et al., 2014) with the default parameters. This process removed low-quality bases, filtered out short reads, and eliminated any contaminating Illumina sequencing adapters. The genome of channel catfish, *I. punctatus*, was obtained from the NCBI (assembly Coco_2.0; accession #GCA_001660625.3), and QC reads were aligned to it using the STAR aligner (Dobin et al., 2013), with options of 2-pass mapping and the overhang length set to 149. QC of the resultant BAM files was performed using RSeQC modules (Li et al., 2009; Wang et al., 2012; Wang et al., 2016) to assess alignment quality and obtain quality scores, such as transcript integrity numbers (TINs). At this stage, one sample (B1) was removed from further analyses. Sample B1 (a high-frass liver sample) was noted as a technical outlier as the integrity score was quite low (TIN = 18.52). QC BAM files were then used to generate a gene-level count data matrix for all samples via the HTSeq software (Anders et al., 2015). In HTSeq, the quantification level was set to feature type exon and grouped by parent attributes; the strand specificity was set to non–strand specific; and the overlap mode was set to union. The R package DESeq2 (Love et al., 2014) was then used to generate differentially expressed genes (DEGs) in pairwise comparisons of low- or high-frass samples as compared to the control, no frass (baseline) samples. Principal component analyses were performed on the data, and an additional sample (B2) was removed from further analyses. Sample B2 (a low-frass liver sample) was also noted as an outlier since it had a first principal component score >>>1. Finally, histograms of *p*-values were examined and adjusted using the fdrtool (Strimmer, 2008). The resultant DEG lists (Supplementary Table S1) were considered significant if the adjusted *p*-value was less than 5% and the fold change was greater than 2-fold up- or downregulated as compared to the control (*p*-adj <0.05 and FC > 2).

### 2.6 Quantitation of channel catfish innate and adaptive immune gene expression

Innate and immune gene-specific primers (Table 2) were utilized to independently evaluate channel catfish immunity in response to frass inclusion via reverse transcription quantitative PCR (RT-qPCR). Each total RNA sample was assessed by using spectrophotometry (Bio-Tek Cytation 1, Agilent Technologies, Palo Alto, CA) and Agilent 2100 Bioanalyzer with RNA integrity numbers (RINs) > 8 before reverse transcription. Then, cDNA synthesis was performed using the LunaScript^®^ RT SuperMix Kit (New England Biolabs, Ipswich, MA, United States). Reactions contained 4.0 μL of LunaScript RT SuperMix (5X) and template RNA (200 ng), and the volume was adjusted using nuclease-free water to 20 μL. As a control, to rule out the presence of DNA in the extracted sample, no-RT reactions were prepared for each of the samples along with no template controls (negative control). Reaction conditions for cDNA synthesis included primer annealing at 25°C for 2 min, cDNA synthesis at 55°C for 10 min, and heat inactivation at 95°C for 1 min. cDNA was kept at −20°C after transcription until RT-qPCR.

**TABLE 2:** List of RT-qPCR primers.

Gene	Forward primer (5′-3′)	Reverse primer (5′-3′)	Reference
**CD4**	CCT​CTG​CGA​ACC​CAT​CTT​CA	AGG​CAG​GTC​CAG​ATT​CTT​GC	Xu et al. (2016)
**IL-1β type a**	AAA​AAT​GGC​CAG​CCT​GTA​TG	CAG​CCC​GGG​TAT​TTA​ACT​GA	
**IL-1β type b**	GCC​TCT​TAG​TAT​GCG​CCA​AG	AAC​CTT​GTC​TTG​CAG​GCT​GT	
**Complement factor D**	AGG​CAG​AGG​ACA​AAA​AGC​AA	TGG​CTG​ACT​TAG​CTG​CCA​AT	
**Complement factor C3**	AGT​TGA​ATA​CCG​CTG​CCA​AC	CTC​TCC​ATG​CGC​TGA​GTA​CA	
**MHC class II**	CTG​AGG​AAC​GGG​AAG​GAG​AT	CAG​ATG​GGA​GTG​GAC​CTG​AT	
**Interferon γ**	CAG​CAG​TGA​CTT​CAG​CCA​AA	GCC​TCA​GAG​TAC​GCC​ATC​AT	
**TLR1**	AGC​CAA​AGA​AAT​GCC​AAC​TG	TGA​AGT​CTC​GTT​CGT​GGT​GA	
**TLR9**	GGA​GGA​ACG​GGA​CTG​GAT​AC	AAG​CAC​AGC​CAC​CCT​GAT​TA	
**IgM**	TGT​GTG​TGT​GTG​TGT​GTG​TGT​T	CGG​ATG​TCT​TGG​CTT​GTT​G	
**IL-17**	TGG​TTG​CTC​AGG​CTG​CTC​CTT	ACG​CCA​GCT​TGA​TGT​CAT​GTT​CC	Wang et al. (2014)
**18S**	GAG​AAA​CGG​CTA​CCA​CAT​CC	GAT​ACG​CTC​ATT​CCG​ATT​ACA​G	Small et al. (2008)
**β2M**	AAG​GGA​TGG​AAG​TTT​CAT​CTG​ACC	GGA​ATG​AAG​CCC​AGG​AGG​TTT​AC	
**β Actin**	CCC​ATC​TAT​GAG​GGT​TAT​GCT​CTG	GCT​CGG​TCA​GGA​TCT​TCA​TCA​G	
**TLR3**	TTG​CAG​CTG​TGA​GAG​CAT​TC	AGT​GCA​CCA​GGA​AGG​CTA​GA	Pridgeon et al. (2010)
**TLR5**	TTG​GAA​GCG​CTA​CAA​ATC​CT	ACC​CGG​AGG​TTG​AAT​AAT​CC	
**TLR20A**	CAC​CTC​TCT​GGG​ACT​GGT​GT	GCT​CAT​CTT​TCC​CGC​AGT​AG	
**TLR21**	TTC​CTC​TGC​AGT​GAG​TGG​TG	TGT​GTC​CAG​AAC​AGC​TCC​TG	
**LEAP**	CCT​TTG​GAG​AAT​CAT​GGG​TAC​TAA	GCA​GTG​TCC​TTT​CCT​GCA​TA	This study
**Hepcidin**	GTTGGCGAAGGAGACGAG	ACC​CAC​AGC​CTT​TAT​TCT​TAC​A	
**TNF α**	GCT​GCA​ATC​AGA​ACG​ACT​AGA	GGT​CCG​TCC​ACA​TCC​AAT​AC	

Using a LightCycler^®^ 480 System (Roche Diagnostics, Indianapolis, IN), RT-qPCR was performed to assess the expression level of systemic (head kidney and liver) and mucosal (gill and intestine) immunity by checking differential regulation of innate and adaptive immune genes. Fourteen innate immune genes such as six innate receptors (TLR1, TLR3, TLR5, TLR9, TLR20A, and TLR21), five proinflammatory cytokines (TNFα, IFN-γ, IL-17, IL-1β type a, and IL-1β type b), two chemokines (complement factor D and complement factor C3), and one antimicrobial peptide (hepcidin) were used for this study. Four adaptive immune genes were used for this study, that is, an immunoglobulin (IgM), two major histocompatibility class I (CD4 and β2M), and major histocompatibility class II (MHC class 2) genes. The Luna Universal qPCR Master Mix was used for RT-qPCR in 10 μL reactions. Each reaction included 5 μL of Luna Universal qPCR Master Mix (2X), 0.5 μL of forward primer (1 μM), 0.5 μL of reverse primer (1 μM), 2 μL of nuclease free water, and 2 μL of cDNA diluted 1:10 with nuclease-free water. Reactions were carried out in triplicate under the following conditions: 95°C for 15 s, followed by 45 cycles at 95°C for 15 s, 60°C for 30 s, followed by melting curve analysis. Reaction and cycling conditions were prepared following the manufacturer’s instructions (NEB). The samples were run in parallel with two reference genes, 18S and EF1α, for normalization.

Relative gene expression was calculated using the 2^−ΔΔCT^ method (Livak and Schmittgen, 2001; Pfaffl, 2001), normalizing with the geometric average of the reference gene relative to the controls. Statistical assessment was performed using the IBM SPSS 20 software (SPSS Inc., Chicago, IL). Data were evaluated by the analysis of variance (ANOVA) to determine differences in means among different groups and then further analyzed *post hoc* using Tukey’s multiple comparison test. Differences of means among the groups were considered statistically significant when *p* < 0.05. Data were collected and analyzed, and all graphs of immune genes were plotted using the GraphPad Prism 5 software (Boston, MA).

## 3 Results

### 3.1 Global gene expression analyses of channel catfish tissues after being fed either a low-frass (50 g/kg) or high-frass (300 g/kg) experimental diet

**FIGURE 1:**
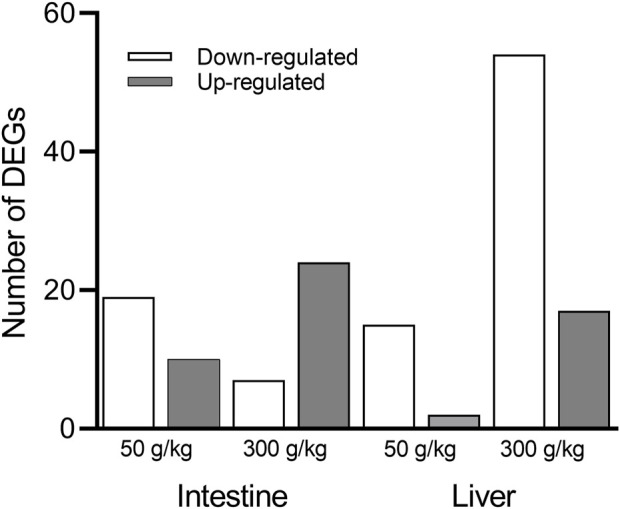
Number of significant DEGs identified between the control (0 g/kg) and the low-frass (50 g/kg) and high-frass (300 g/kg) supplemented diets. The downregulated DEGs (white bars) and upregulated DEGs (gray bars) are shown for the intestine and liver (*p*-adj <0.05; fold change >2).

The liver and intestine of individual channel catfish fed with different frass diets (50 and 300 g/kg frass) or control diet (0 g/kg frass) were used to build and sequence mRNA libraries. After the raw RNA sequencing data were processed and aligned with the channel catfish genome, sequence libraries from the 50 and 300 g/kg frass for the intestine and liver were compared to the basal-level control intestine or liver (0 g/kg frass) using the cutoff criteria (*p*-adj <0.05, fold change >2). There were 19 downregulated and 10 upregulated genes among the 29 DEGs identified in the low-frass (50 g/kg) intestine, and seven downregulated and 24 upregulated genes among the 31 DEGs identified in the high-frass (300 g/kg) intestine (Figure 1) samples. There were 17 DEGs, which included 15 downregulated and two upregulated in the low-frass (50 g/kg) liver samples. There were 54 downregulated and 17 upregulated genes among the 71 DEGs identified in the high-frass (300 g/kg) liver samples (Supplementary Material S1).

**FIGURE 2:**
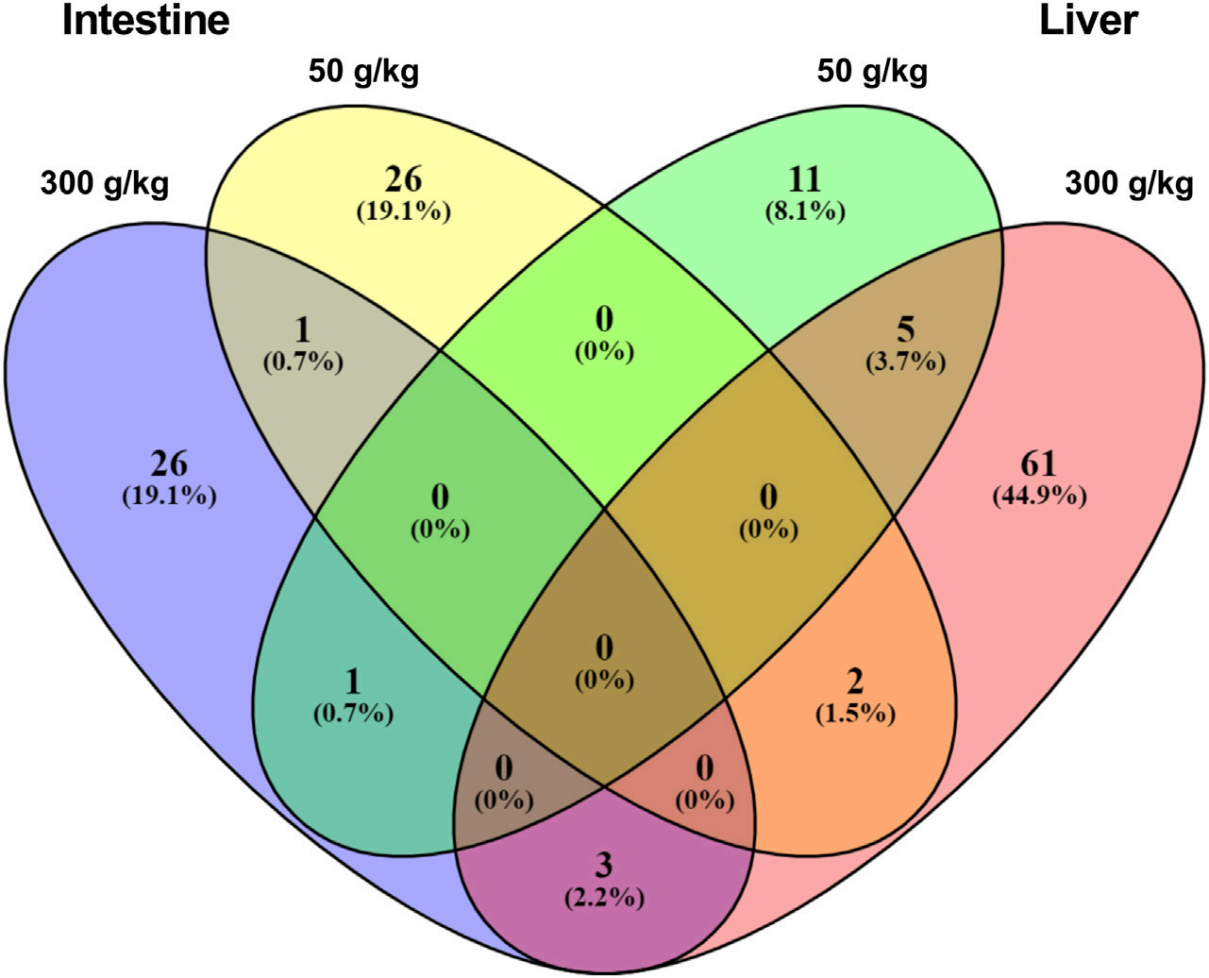
Venn diagram showing unique DEGs among the low-frass (50 g/kg) and high-frass (300 g/kg) supplemented diets in the intestine and liver.

We next sought to understand the relationship between the DEGs identified among the different experimental frass diets and their respective tissues. For this analysis, we identified the gene expression interactions through visualizing the DEGs in a Venn diagram (Figure 2 and Supplementary Material S1). There were five DEGs that were co-expressed in the low- and high-frass liver, whereas there was only one co-expressed between the low- and high-frass intestine, and there were no common DEGs among the all frass diets. However, there were three DEGs that were shared among the intestine and liver high-frass diets, two DEGs among the intestine low-frass and liver high-frass diets, and one DEG among the intestine high-frass and liver low-frass diets. Hence, only ∼3% of DEGs were common among the liver and intestine in the frass diets and most of the DEGs in each low-frass and high-frass diets in the liver and intestine were unique to the groups when compared with the control diet (Supplementary Material S1).

Several upregulated DEGs that represent different innate immune receptors and effector molecules such as toll-like receptor 5 were identified in the low-frass (FC = 4.6) and high-frass (FC = 6.1) intestine; apolipoprotein A1 (FC = 4.1) in the low-frass intestine; and C-type lectin (FC = 2.4), complement factor H (FC = 4.7) and lysozyme (FC = 13.4) in the high-frass intestine. In the low-frass liver, there was hepcidin (FC = 11.9) that was upregulated in the low-frass liver, and in the high-frass liver, there was lysozyme (FC = 3.0).

Several interferon or interferon-like proteins were downregulated in the liver, as well as genes associated with adaptive immunity such as major histocompatibility complex class I (FC = −35.9) and immunoglobulin-like V-region gene (FC = −9.3) in the low-frass intestine. Alternatively, glucose-6-phosphatase catalytic subunit 1a (FC = 9.0) and myostatin (FC = 4.7) were upregulated in the low-frass intestine, which correspond to the regulation of cell proliferation, apoptosis, and the regulation of skeletal muscle growth in the channel catfish.

### 3.2 Immune gene expression in channel catfish

Based on the RNA sequencing results of the low-frass (50 g/kg) and high-frass (300 g/kg) diets, we next performed a more thorough analysis of innate and adaptive gene expression using both systemic (head kidney and liver) and mucosal (gill and intestine) tissues from all the frass-supplemented diets (50, 100, 200, and 300 g of frass per kilogram of feed) as compared to the control, no frass inclusion diet (Yildirim-Aksoy et al., 2020a).

#### 3.2.1 Expression of innate immune receptors among different tissues

**FIGURE 3:**
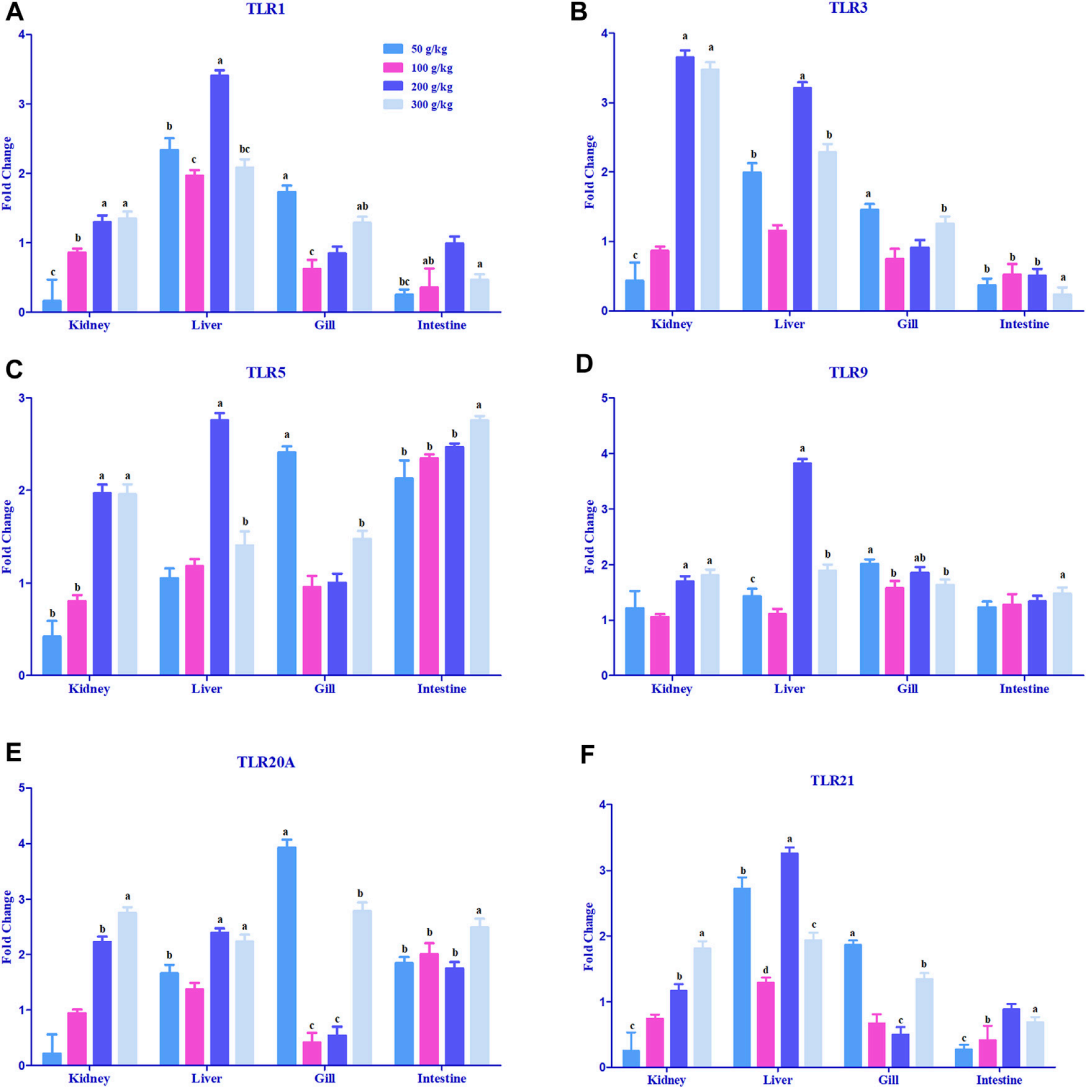
Relative expression of toll-like receptors: TLR1 **(A)**, TLR 3 **(B)**, TLR5 **(C)**, TLR 9 **(D)**, TLR20A **(E)**, and TLR 21 **(F)** in the head kidney, liver, gills, and intestine of channel catfish fed with frass-supplemented diets (50–300 g/kg). Data are presented as mean ± SE, and multiple reference genes were used to normalize with the target gene (n = 4). TLR gene expression among the frass diets (50–300 g/kg) was significantly upregulated when compared to the control (0 g/kg). Different alphabets indicate the significant differences between frass-supplemented diets (*p* < 0.05).

A significant difference in the expression levels of various toll-like receptor genes was observed in the gill, intestine, head kidney, and liver samples when compared to the control tissues (0 g/kg); however, only some of the tissues demonstrated a threshold of >2-fold change (Figure 3). The expression of TLR1 was mostly upregulated in the liver with a 2-fold increase in expression among the frass diets (Figure 3A). TLR3 expression was largely expressed in the head kidney (3.5-fold) with higher frass concentrations and (2- to 3-fold) across the liver (Figure 3B). TLR5 was the only >2-fold upregulated among the liver and gills in one to two of the frass diets (Figure 3C). The intestine showed >2-fold upregulation among all the frass diets. The TLR9, TLR20A, and TLR21 genes were mostly >2-fold upregulated among the high-frass (200 and 300 g/kg) diets in the liver (Figures 3D–F). The TLR20A gene had >2-fold expression among the different frass diets in the head kidney, gills, and intestine.

#### 3.2.2 Expression of proinflammatory cytokines in different tissues

**FIGURE 4:**
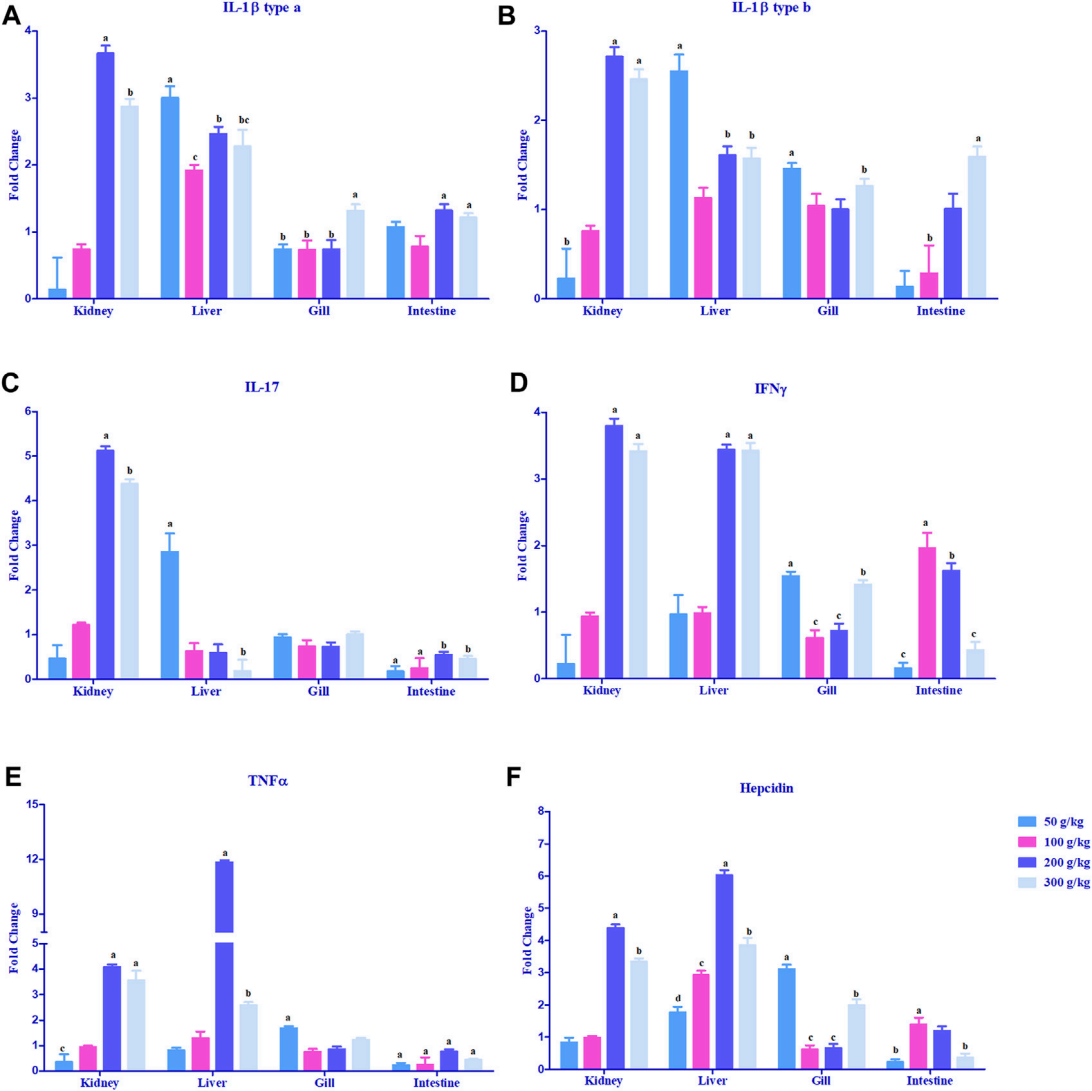
Relative expression of proinflammatory cytokines: IL-1β type a **(A)**, IL-1β type b **(B)**, IL-17 **(C)**, TNFα **(D)**, and IFN-γ **(E)**, and antimicrobial peptide and hepcidin **(F)** in the head kidney, liver, gills, and intestine of channel catfish fed with frass-supplemented diets (50–300 g/kg). Data are presented as mean ± SE, and multiple reference genes were used to normalize with the target gene (n = 4). Gene expression among the frass diets (50–300 g/kg) was significantly upregulated when compared to the control (0 g/kg). Different alphabets indicate the significant differences between frass-supplemented diets (*p* < 0.05).

The expression of cytokine genes, IL-1β type a and IL-1β type b, was upregulated >2-fold in the head kidney and liver mostly with the high-frass (200 and 300 g/kg) diets with an exception in the liver where the low-frass (50 g/kg) diet also exceeded the 2-fold change difference (Figures 4A, B). IL-17 and IFN-γ had a similar pattern in the head kidney with >2-fold upregulation mostly among the high-frass (200 and 300 g/kg) diets, whereas in the liver, IL-17 was most abundantly expressed with the low-frass diet and IFN-γ was upregulated among the high-frass (200 and 300 g/kg) diets (Figures 4C, D). The gills and intestine had significant upregulation when compared to the control tissues (0 g/kg), but neither tissue demonstrated a threshold of >2-fold upregulation. TNFα was upregulated >2-fold in the head kidney with high-frass (200 and 300 g/kg) diets, while the liver had greatly overexpressed expression in the 200 g/kg diet and also significant expression in the 300 g/kg diet (Figure 4E).

#### 3.2.3 Expression of hepcidin and complement genes in different tissues

**FIGURE 5:**
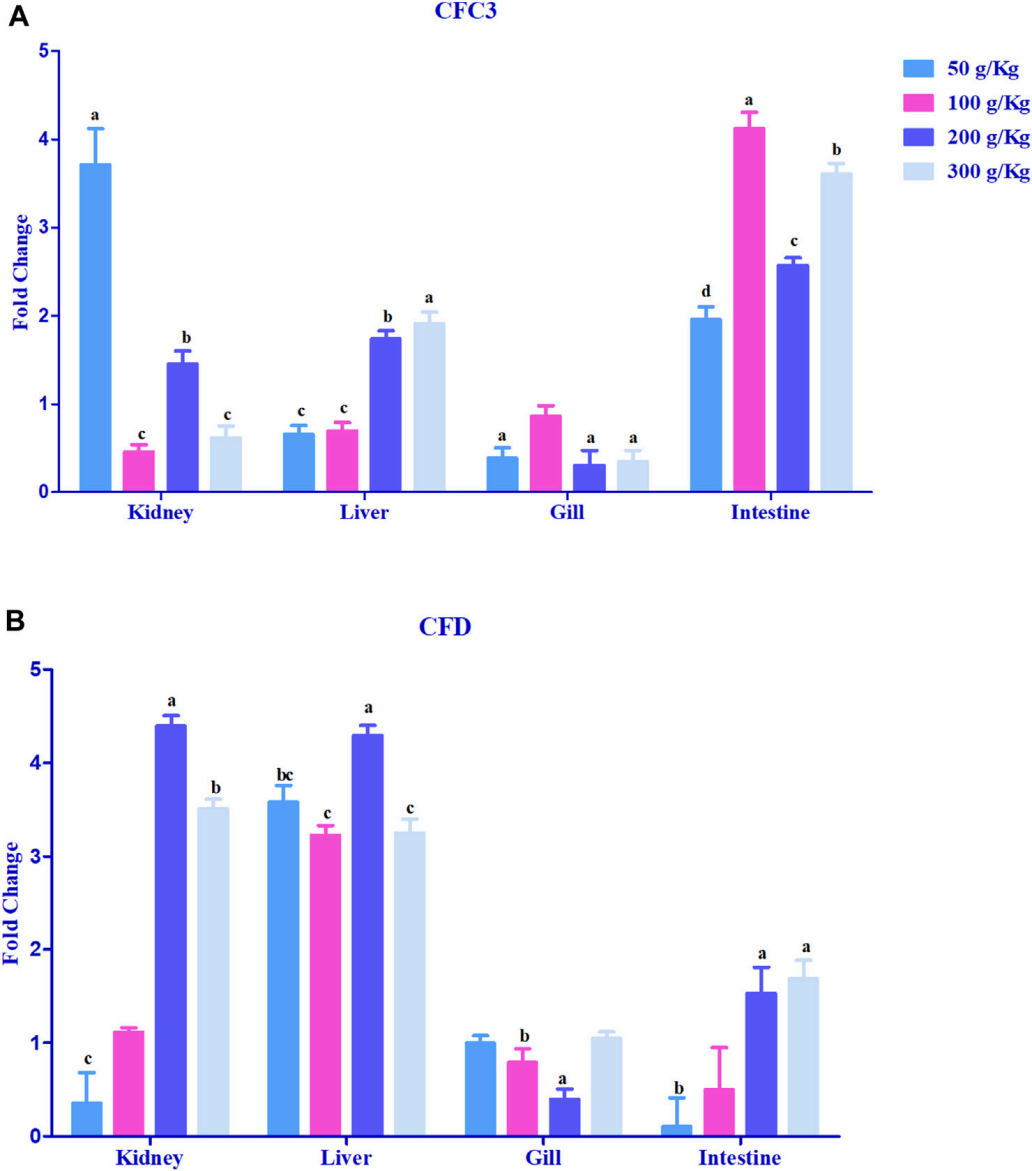
Relative expression of complementary factor C3 **(A)** and complement factor D **(B)** in the head kidney, liver, gills, and intestine of channel catfish fed with frass-supplemented diets (50–300 g/kg). Data are presented as mean ± SE, and multiple reference genes were used to normalize with the target gene (n = 4). Different alphabets indicate the significant differences between frass-supplemented diets (*p* < 0.05).

Hepcidin gene expression was upregulated >2-fold in the head kidney with high-frass (200 and 300 g/kg) diets and in the liver among the 100–300 g/kg frass diets (Figure 4F). The gills had >2-fold upregulation with the 50 g/kg frass diet, and the intestine demonstrated no >2-fold upregulation. Complement factor C3 was upregulated >2-fold in the intestine and in the head kidney with >2-fold upregulation with the low-frass diet (Figure 5A). Complement factor D was predominantly upregulated >2-fold in the head kidney with high-frass diets (200–300 g/kg) and in the liver among all frass diets (Figure 5B).

#### 3.2.4 Expression of adaptive immune genes in different tissues

**FIGURE 6:**
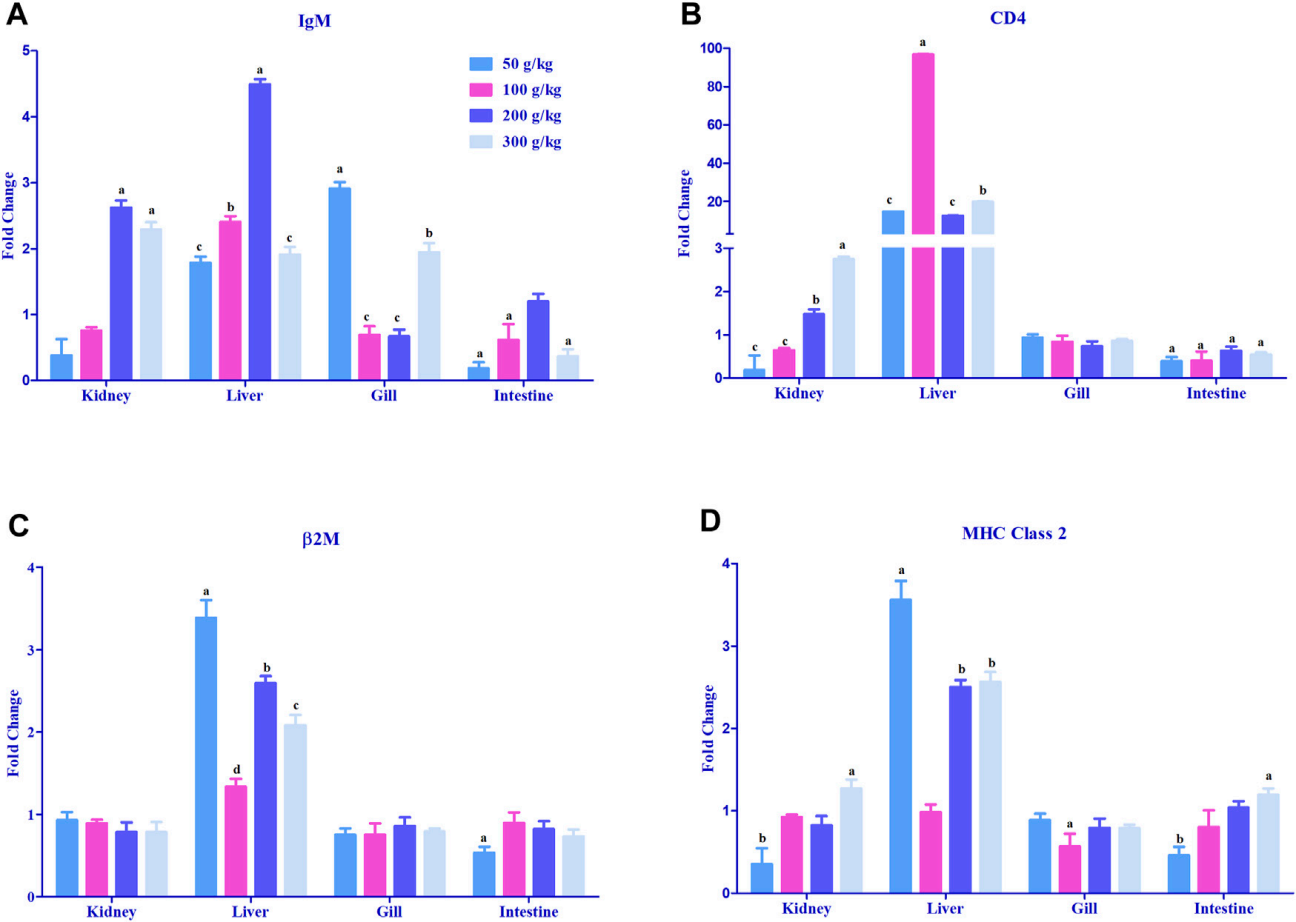
Relative expression of adaptive immunity genes: IgM **(A)**, CD4 **(B)**, β2M **(C)**, and MHC class 2 **(D)** in the head kidney, liver, gills, and intestine of channel catfish fed with frass-supplemented diets (50–300 g/kg). Data are presented as mean ± SE, and multiple reference genes were used to normalize with the target gene (n = 4). Different alphabets indicate the significant differences between frass-supplemented diets (*p* < 0.05).

The adaptive immune-related genes (IgM, MHC class 2, β2M, and CD4) showed significant differential expressions in the gills, head kidney, and liver. The IgM gene was mostly >2-fold upregulated with the high-frass (200–300 g/kg) diets in the head kidney and with the 100–200 g/kg frass diets in the liver. The gills had >2-fold upregulation in the 50 g/kg frass diet, and the intestine demonstrated no >2-fold upregulation (Figure 6A). CD4 expression was upregulated >20-fold almost exclusively in the liver tissue among all frass diets, and >2-fold upregulation was identified in the head kidney with the 300 g/kg diet (Figure 6B). In liver, frass diets of 50, 200, and 300 g/kg were the only ones with >2-fold upregulation of the β2M gene (Figure 6C). Again, the liver was the site for >2-fold upregulation of the MHC class 2 gene among the 50, 200 and 300 g/kg frass diets (Figure 6D).

### 3.3 Validation of RNA-seq with RT-qPCR

RNA-seq and RT-qPCR values were compared for the intestine with the low- and high-frass diets (50 and 300 g/kg). TLR5, complement factor D, and complement factor C3 all had similar fold change values (Table 3).

**TABLE 3:** Comparison of RNA-seq and RT-qPCR results in the intestine with either 50 or 300 g/kg frass in the diet.

Feature	Description	Fold change	
		50 g/kg	300 g/kg
		RT-qPCR (RNAseq)	RT-qPCR (RNAseq)
**NM_001200229.1**	Toll-like receptor 5	2.1 (4.6)	2.7 (6.1)
**NM_001257116.1**	Complement factor D	0.1 (ND^a^)	1.7 (1.7)
**XM_017457539.3**	Complement C3	1.9 (ND^a^)	3.6 (3.0)

^a^ No data.

## 4 Discussion

Insects are composed of essential amino acids like that in fish meal. BSF (*H. illucens*) larvae are one of the most promising insect species for use in aquaculture feed (Yildirim-Aksoy et al., 2020a; Yildirim-Aksoy et al., 2020b; Yildirim-Aksoy et al., 2022; Mohan et al., 2022). Although substrates for BSFL often vary greatly in their nutritional components, frass generated from larvae fed on distillers’ dried grains contain enough protein to serve as an animal feed source. Previous work in our lab has demonstrated the nutritional benefit of frass-supplemented diets in channel catfish with increased growth performance and an increase in overall feed intake and palatability (Yildirim-Aksoy et al., 2020a). In the current study, we investigated the effect of BSFL frass diets on gene expression among different systemic and mucosal tissues in the channel catfish.

A previous work has shown that proinflammatory genes such as toll-like receptor (TLR) signaling pathway enhance disease resistance in teleost fish (Chunyan et al., 2023). An analysis of rainbow trout transcriptome data revealed no activation of the TLRs after 30 days (Chunyan et al., 2023), whereas a previous study in rainbow trout had shown significant upregulation of the TLR genes in the liver and spleen after the use of probiotics (Dang et al., 2022; Chunyan et al., 2023). Different TLRs on the surface of host cells are honed to react quickly to pathogens, and the different probiotic strains that are species specific likely carry out their “probiotic effect” by modulating host gene expressions, which could lead to a more robust innate immune response (Dawood et al., 2020; Tippayadara et al., 2021).

In the current study, TLR5 was upregulated >2-fold in the intestine in both the 50 and 300 g/kg frass diet RNAseq transcriptome data and in the quantitative PCR analyses of all the frass diets in the intestine. TLR1, TLR3, and TLR21 peaked in the head kidney and liver of channel catfish fed with high-frass diets (200–300 g/kg). To date, there are few studies evaluating the gene expression of innate immune receptors in channel catfish fed with BSFL frass diets. BSFL-supplemented diets consist of chitin and are tightly bound to β-glucan to form chitin–glucan complexes, which are specifically used by probiotic bacteria such as *Lactobacillus* and *Bacillus* for their development in fish intestines (Balcázar et al., 2006; Dawood et al., 2020; Wang et al., 2023). Immunostimulants work by recognizing certain molecular structures that the host cells have discovered. Pathogen-associated molecular patterns (PAMPs) are repetitive structures that are seen in a variety of microorganisms. PAMPs that are present in or on microorganisms during a natural infection, be it on bacteria, viruses, or fungi, are recognized by certain receptors on or in the host cells that are being infected. The receptors, which may be membrane-bound or cytosolic, are collectively referred to as pathogen recognition receptors (PRRs), and they comprise toll-like and RIG-I receptors among others. Following the activation of the innate immune response by these molecules, proinflammatory cytokines may be produced and released (Dawood et al., 2020).

Proinflammatory cytokines and chemokines are essential for cell proliferation, apoptosis, growth, and development where interleukin-10, an anti-inflammatory cytokine, and inflammation mediators NFκB and MYD88 are useful markers of inflammation when evaluating new ingredients in feed formulations (Zarantoniello et al., 2018; Zarantoniello et al., 2020a; Zarantoniello et al., 2020b; Zarantoniello et al., 2022; Ratti et al., 2023). In the current research, channel catfish fed with diets containing BSFL frass had substantially elevated levels of IL-1β type a, IL-1β type b, IL-17, TNFα, and IFN-γ, generally in the head kidney, when fed with higher frass diets and among all frass diets in the liver. The cytokines were much less expressed in the intestine and gills of channel catfish fed with the frass diets, which is comparable with studies with BSFL meal in trout and swamp eel (Fawole et al., 2021; Kumar et al., 2021; Fischer et al., 2022). Among teleost fish, gene expression responses have been shown to be variable, yet appear to be species- and stage-specific as well as connected to the dietary inclusion amounts of BSFL meal. For instance, among the several developmental stages in zebrafish, different immune response markers can be expressed in the gut, for example, in adult zebrafish, these markers (*il1b*, *il6*, and *tnfa*) were not significantly expressed in 25% and 50% full-fat BSF prepupae meal (Zarantoniello et al., 2019). Dietary inclusion of 50, 60, or 100% BSFL meal did not adversely impact Atlantic salmon pre-smolt that had no significant gene expression (*il4*, *tgf1*, *il10*, *ifn*, *il8*, and *myd88*) when compared to the control (Li et al., 2019) and seawater phase (*il1b*, *il17a*, *myd88*, *il8*, *il4*, *mhcl*, *il10*, *ifn*, *tgf*, *cd8*, and *foxp3*) with no significant expression of cytokines (Li et al., 2020). Immune gene expression was activated in the intestines of both larval and juvenile zebrafish (*IL1b*, *IL10*, *IL6*, and *tnfa*) (Zarantoniello et al., 2018; Zarantoniello et al., 2019; Zarantoniello et al., 2020a; Zarantoniello et al., 2020b; Zarantoniello et al., 2021; Zarantoniello et al., 2022), juvenile rainbow trout (*IL10*, *tnf*, and *tlr5*) (Cardinaletti et al., 2019), both the proximal and distal intestines of Atlantic salmon (*cd3* and *foxp3*) (Li et al., 2019), and in the distal intestine of rainbow trout (*il1b* and *tlr1*) (Mohan et al., 2022; Ratti et al., 2023), which supports our research findings in the channel catfish. As an alternative to fish meals, BSFL-supplemented meals had no detrimental effects on growth and dramatically increased the cytokine expression in the gut of seabass (Hender et al., 2021). In fish, TNFs regulate apoptosis, lipid metabolism, inflammation, and organ regeneration, among other well-documented physiologically significant roles (Yaoguo et al., 2021). The 25% inclusion of BSFL meal diets showed a 9.2-fold increase in IFN-γ relative to control in the Atlantic salmon (Weththasinghe et al., 2021). By producing a variety of iNOS, superoxide anions, and oxygen and nitrogen radicals, TNFα and IFN-γ were upregulated, which in turn activated macrophages (Muñoz-Carrillo et al., 2018). In this study, TNFα and IFN-γ were greatly induced in channel catfish head kidney and liver when fed with increasing amounts of BSFL frass.

In the present study, adaptive immune genes especially IgM have resulted in decreased expression in the intestine, while the head kidney, liver, and gills show varied upregulation among frass diets. The results of another study using BSFL diets identified decreased IgM expression in the distal intestine of salmon as well (Weththasinghe et al., 2021). β2M and MHC class 2 were also significantly upregulated in all BSFL frass diets in liver tissues. The increased β2M expression in BSFL frass diets lead to the activation of cytotoxic lymphocytes as a preventive defense mechanism against pathogens. β2M and CD4 are an essential part of the adaptive immune response from MHC class I molecules, which are responsible for their structure, correct folding, and cell surface expression (Pikulkaew et al., 2020).

Metabolically, channel catfish fed with frass diets developed better than the control (Yildirim-Aksoy et al., 2020a). This could be associated with upregulated genes in the intestine such as glucose-6-phosphatase, which corresponds to the regulation of cell proliferation, and myostatin in the intestine, which regulates skeletal muscle growth. Previous work have demonstrated that increased myostatin leads to muscle growth in catfish (Gregory et al., 2004; Weber et al., 2005; Khalil et al., 2017; Coogan et al., 2022).

Based on these results, meal composition may have an impact on the activity of digestive enzymes in the intestine, which could also be related to mucosal immunity in channel catfish. The upregulated genes associated with cell proliferation and downregulation of several chemokines in the intestine could be involved in nutrient absorption. Previous studies analyzing the transcriptome of probiotic-fed fish have shown a shift in the functional effect of signaling pathways, immune-related pathways, protein digestion and absorption, and starch and sucrose metabolism (Tacchi et al., 2011; Tacchi et al., 2012; Gonçalves et al., 2017; Dawood et al., 2020; Chunyan et al., 2023). Additionally, we observed the upregulation of metabolic pathways that include glucose-6-phosphatase in the intestine of frass-fed fish, which is on par with the studies related to probiotic-incorporated feed (Chunyan et al., 2023; Wang et al., 2023).

## 5 Conclusion

Transcriptome analyses of channel catfish fed with diets formulated with the inclusion of frass from BSFL showed that a series of metabolic and immune-related genes were differentially regulated after being fed either a low- or high-frass diet for 10 weeks. Further analysis of both systemic and mucosal tissues using quantitative PCR revealed an upregulation of multiple innate and adaptive immune genes with various levels of frass supplementation. Overall, these data suggest that the activation of multiple immune-related genes after being fed BSFL frass may improve pathogen resistance in channel catfish, of which further study is warranted.

## Data Availability

The RNA sequencing datasets generated for this study can be found in the NCBI Gene Expression Omnibus (GEO) repository and can be accessed under the accession number GSE231874. All other data that support the findings of this study have been included in the manuscript and Supplementary Materials.
